# Mineral Waters

**Published:** 1887-06

**Authors:** 


### Editorial.

### MINERAL WATERS.

The custom of annually visiting and sojourning for a longer or shorter time at some health resort has become so common that, perhaps, few of our readers will not be consulted within the ensuing month by persons who wish professional guidance in the selection of the place best suited to their condition. Intelligent advice on this subject is not easily given.

There are probably a thousand springs of greater or less repute within easy access of Atlanta; the relative value of each of them is to be judged by their chemical composition and by ascertained results of their use for weeks or months. Unfortunately decisive evidence upon these points has not been obtained.

Accurate analysis of the water of very few of the springs has been published, but in a general way it is known that in most of them the mineral elements which predominate are either iron, sulphur, carbonic acid, magnesia or lime in varying proportions, with traces of other agents of uncertain therapeutic value. The temperature, ranging from 52° to 110°, is easily ascertained.

Upon these vague data, in the absence of verified scientific analysis, an opinion is predicated, and is necessarily inconclusive; the physician who gives and the patient who receives it regard it as an experiment to be tested by results. The wide publication of accurate analysis of the waters of the many springs now competing for patronage ought to enable practitioners to adapt the remedies compounded by nature to the varying condition ot their patients with a certainty as great as that they feel in prescribing those compounded by the druggist.

In the lamentable absence of this, the public must continue, as it has done for many centuries, to estimate the value of these waters by their general reputation.

From the earliest ages the peculiar taste, odor or temperature of different fountains attracted public notice; experiments with them were followed by cures of various diseases. Self-interest, superstition and love of the marvelous magnified the number until magical and divine virtues were attributed to many of them, and from the time of Greece and Rome, they have been chosen places of hopeful and fashionable resort.

It cannot in this day be denied that great benefit, and often cures, follow—in fact are wrought by a residence at any of them.

Interested proprietors may exaggerate the number; change of climate, habits, society, regimen and relaxation from the cares of business may be co-operative influences, but no fact is more universally admitted than that the annual visit to the springs is conducive to vigorous health and to prolonged life.

In the existing state of information as to the exact composition of the various waters now in use, and in the absence of exact observation of their effect in individual cases, it is difficult to choose between them.

At each and all of them the visitor is overwhelmed by a cloud of witnesses and certificates of wonderful cures of every imaginable disease. Whatever the composition of the water, the good results appear to be the same in like cases of disease and almost lead to the conclusion that to the well-known alterative action of the water alone, taken abundantly for a length of time, is attributable the good effects so generally conceded.

While the evidence accessible shows that the benefit of a course at any of the watering places is about equal in all classes of diseases, regardless of the ascertained or conjectured composition of the water, it is hazardous to express a decided opinion of the superior virtues of any one of them; but the consensus of medical and popular opinion of their general efficacy is so unanimous that it may be considered safe and proper to advise patients who are in circumstances to undertake such pilgrimages to do so, "to stand not upon the order of their going, but go at once."

